# The cyclic peptide labaditin does not alter the outer membrane integrity of *Salmonella enterica* serovar Typhimurium

**DOI:** 10.1038/s41598-019-38551-5

**Published:** 2019-02-13

**Authors:** Simone C. Barbosa, Thatyane M. Nobre, Diogo Volpati, Eduardo M. Cilli, Daniel S. Correa, Osvaldo N. Oliveira

**Affiliations:** 1 São Carlos Institute of Physics, University of São Paulo, CP 369, 13560-970 São Carlos-SP, Brazil; 2 Sol Voltaics AB, 223 63 Lund, Sweden; 3 Universidade Estadual Paulista (UNESP), Institute of Chemistry, 14800-060 Araraquara-SP, Brazil; 4 Nanotechnology National Laboratory for Agriculture (LNNA), Embrapa Instrumentação, 13560-970 São Carlos, SP Brazil

## Abstract

Antimicrobial peptides are a promising class of new antibiotics with the ability to kill bacteria by disrupting their cell membrane, which is especially difficult for Gram-negative bacteria whose cell wall contains an outer layer of lipopolysaccharides (LPS). Here we show that the cyclic decapeptide Labaditin (Lo), with proven activity against the Gram-positive *Staphylococcus aureus* and *Streptococcus mutans*, is not able to kill the Gram-negative *Salmonella enterica* serovar Typhimurium (*S.e*.s. Typhimurium). We found that Lo induced significant changes in the surface pressure isotherms of Langmuir monolayers representing the *Salmonella enterica* serovar Typhimurium inner membrane (*S.e*.s. Typhimurium IM), and caused leakage in large unilamellar vesicles made with this IM lipid composition. On the basis of these results one should expect bactericidal activity against *S.e*.s. Typhimurium. However, Lo could not interact with a monolayer of LPS, causing no significant changes in either the surface pressure isotherms or in the polarization-modulated infrared reflection absorption spectra (PM-IRRAS). Therefore, the failure of Lo to kill *S.e*.s. Typhimurium is associated with the lack of interaction with LPS from the outer bacteria membrane. Our approach with distinct monolayer compositions and combined techniques to investigate molecular-level interactions is useful for drug design to fight antibiotic-resistant bacteria.

## Introduction

*Salmonella* is a Gram-negative bacterium with bacillus shape from the Enterobacteriaceae family. *Salmonella enterica* comprises more than 2600 different serovars classified into typhoidal and nontyphoidal (NTS). NTS usually cause gastroenteritis with occasional secondary bacteremia^1^, but the typhoidal counterparts, which are adapted to humans and do not occur in other animals, typically cause severe illnesses such as typhoid fever (Typhi), paratyphoid fever (Paratyphi), and food poisoning^2^. Treating infections caused by Gram-negative bacteria is challenging due to the molecular structure of their membrane, made up of an inner membrane (IM) and an outer membrane (OM)^3^. IM is a symmetrical bilayer essentially composed of phospholipids, which in *S.e*.s. Typhimurium include phosphatidylethanolamines (PE), phosphatidylglycerols (PG), and cardiolipin (CL)^4,5^. OM is an asymmetric bilayer containing glycerophospholipids (GPL), lipopolysaccharides (LPS), porins, and other specific uptake channels^6^. The LPS outer layer comprises three regions: lipid A (a glucosamine-based phospholipid), an oligosaccharide core, and O-antigen^7^. Its complex structure serves as a barrier, yielding a low cell permeability to many drugs.

To be effective against Gram-negative bacteria, antibiotics must be able to either disrupt both inner and outer membranes or cross them via porin channels, which are water channels used by hydrophilic small drugs with molecular weight below ~600 Da^6,8–10^. Antimicrobial peptides (AMPs) are promising for use against a broad spectrum of antibiotic-resistant bacteria^11–13^, especially as they are capable of disrupting cell membranes^14–18^. Labaditin (Lo), a cyclic decapeptide, head-to-tail, extracted from *Jatropha multifida* (peptide sequence - VWTVWGTIAG)^19–21^, for instance, has been proven effective against *Staphylococcus aureus*^22^ and *Streptococcus mutans*^20,21^. Both are Gram-positive, formed by a single lipid bilayer surrounded by a bulky layer of peptidoglycan^23,24^.

In both cases, activity was related to Lo ability of forming pores through the membrane since it could not diffuse through porin channels because of its high molecular weight. The challenge is then to find whether Lo could also kill *S.e*.s. Typhimurium since the outer membrane represents a more difficult barrier. In this study we show that Lo is not able to kill the *S.e*.s. Typhimurium. To determine the reasons for this failure we designed a series of experiments using Langmuir monolayers and vesicles, which are performed with the IM lipid composition, and LPS from OM, separately. The monolayer properties are evaluated using surface pressure isotherms, polarization-modulated infrared reflection absorption spectroscopy (PM-IRRAS), and permeability assays were carried out with large unilamellar vesicles (LUVs). The experiments were also performed with the linear analogue of Labaditin, referred to as L_1_, for the purpose of comparison with Labaditin.

## Results and Discussion

### Lack of activity against *S.e*.s. Typhimurium

AMPs normally have broad effectiveness against bacteria^25–28^. Labaditin (Lo) is active against the *S. aureus* ATCC 25923^22^ and *Streptococcus mutans* sp^21^, but its linear analogue L_1_ was not for either of these bacteria. The difference in bactericide activity was attributed to their distinct abilities to disrupt the lipid membrane of *S. aureus* since Lo formed nanotubes to cross the membrane whereas L_1_ could not^22^. In the MIC determination experiments here we observed that neither Lo nor L_1_ were effective against *S.e*.s. Typhimurium. No inhibition was observed for the concentration range from 1000 to 0.5 μg/mL of these peptides. Therefore, one may hypothesize that this lack of activity can be associated with the difficulty of the peptides in disrupting and/or forming pores in the membrane. Because Gram-negative bacteria have two adjacent membranes, an IM and an OM whose outer leaflet is essentially composed of lipopolysaccharides (LPS)^29^, this hypothesis can only be tested by performing experiments to mimic the two membranes. This is what we attempted to do in the present study.

### Peptide insertion in monolayers mimicking *S.e*.s. Typhimurium IM

**Figure 1:**
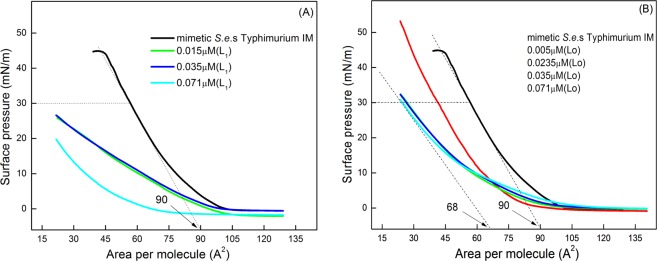
Surface pressure-area isotherms of monolayers mimicking the *S.e*.s. Typhimurium IM (78% DOPE, 4% CL, and 18% DOPG), in the absence and presence of the peptides at distinct concentrations: L_1_ (**A**) and Lo (**B**). The lipid mixture was solubilized in chloroform and spread on the air/water interface. After 15 min for the solvent to evaporate, the peptide solution was injected into the subphase. The surface pressure was measured with the Wilhelmy method and the area per molecule was varied by compressing the monolayer with barriers at a 10 cm^2^.min^−1^ rate. It is assumed that the system is under equilibrium at each surface pressure. The black curves show relatively expanded isotherms for the monolayer of the composition mimicking *S.e*.s. Typhimurium IM. Incorporation of either of the peptides caused the isotherms to shift to smaller areas per molecule.

The simplest procedure to characterize a monolayer at the air/water interface is to obtain the so-called surface pressure isotherm, in which the surface pressure is plotted against the average area occupied by one molecule as the Langmuir trough area is decreased upon compression with the trough barriers. In a previous work, Lo and L1 peptides (0.071 µM) were found to adsorb at the air/water interface to form Gibbs films after being injected in the subphase. L1 yields a more condensed film with higher collapse pressure than for Lo^22^. The black curves in Fig. 1 correspond to the surface pressure isotherm for the Langmuir monolayer obtained by spreading a solution of the lipid composition mimicking *S.e*.s. Typhimurium IM (referred to as “mimetic *S.e*.s. Typhimurium IM”). This composition consisted of 78% DOPE, 4% CL, and 18% DOPG^4,5^. The isotherm for this lipid mixture had no coexistence of phases and a collapse pressure of ~44 mN/m. Addition of either L_1_ or Lo into the subphase (Fig. 1A,B, respectively) affected the lipid film by inducing a shift to smaller areas per molecule. This shift can be attributed to removal of lipids from the interface to the solution in a detergent-like mechanism or because compression disturbed the monolayer integrity. Therefore, judging only by the changes in the surface pressure isotherms, one could predict that Lo and L_1_ both affect the lipid inner membrane.

### PM-IRRAS: peptide effects on *S.e*.s. *Typhimurium* IM Monolayer

**Figure 2:**
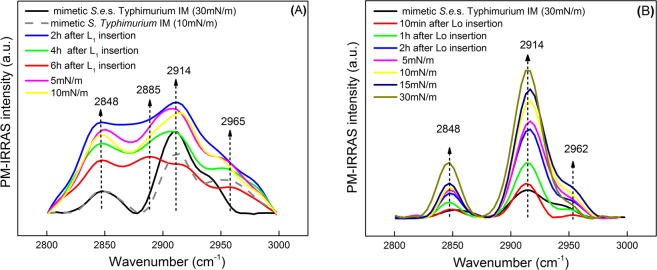
PM-IRRAS spectra taken at the air/water interface for the monolayer mimicking the *S.e*.s. Typhimurium IM (78% DOPE, 4% CL, and 18% DOPG) at 30 mN/m (black curve, (−)). In (**A**) are also shown the spectra for the monolayer incorporating L_1_ under different conditions, namely: at zero surface pressures after 2, 4 and 6 h of injecting the L_1_ solution, and then with the monolayer compressed at 5 and 10 mN/m. In (**B**) similar conditions apply for the *S.e*.s. Typhimurium IM monolayer containing Lo. The changes in condition are in the time after peptide injection and the pressures at which the spectra were taken, as indicated in the figure. The concentration of L_1_ or Lo was 0.071 μM. The region of the spectra shown corresponds to the methylene stretching bands (2800–3000 cm^−1^) present in the lipids.

The adsorption of the peptides on the *S.e*.s. Typhimurium IM monolayer was also monitored by PM-IRRAS, and the spectra taken before and after interaction with L_1_ and Lo are shown in Fig. 2A,B, respectively. The data collection was initially performed with the barriers opened (zero surface pressure) during distinct times after injecting the peptide solution to evaluate the peptide adsorption kinetics. Then, PM-IRRAS spectra were taken at fixed surface pressures (each 5 mN/m) by pausing compression to register the measurements. Special attention is paid to the spectra at 30 mN/m, which is believed to correspond to the pressure in a cell membrane^30–32^. PM-IRRAS is useful to investigate the changes induced on the hydrophobic chains of phospholipid membranes and on the peptide (amide group). Lipid packing in monolayers was studied through the asymmetric and symmetric CH_2_ stretching vibrations at 2914 and 2848 cm^−1^, respectively^33^, seen in the PM-IRRAS spectra at 30 mN/m in Fig. 2. The position of these bands reveals a highly packed monolayer, which should be expected owing to the characteristics of these phospholipids^33^. We can concentrate on these two vibrational signatures to make a first assessment about the effects of adding peptides to the subphase. The addition of Lo into the subphase increased the intensity of the two bands over time and upon compression (Fig. 2B), which confirms Lo insertion in the *S.e*.s. Typhimurium IM monolayer. The intensity increases due to the increasing number of molecules under the incident beam when the area per molecule is reduced, and because the molecules become more ordered upon compression^34^. Through the ratio of relative band intensities, ν_a_(CH_2_)/ν_s_(CH_2_)^34^, it was possible to determine quantitatively the ordering in the hydrocarbon chains within the monolayer. In the presence of Lo, the ratio remained close to 3.7 for all pressures analyzed. It indicates that compression of the monolayer containing Lo does not cause structural changes in the lipid chains up to 30 mN/m. Additionally, no remarkable band shifts were detected, confirming that Lo does not induce a disorganizing effect in the hydrophobic chains^35^. As for L_1_, its insertion induced a slight shift of the asymmetric CH_2_ stretching band shown in Fig. 2A, but the band had low resolution and low intensity, which hampered the data analysis and baseline determination. The ratio of relative band intensities decreased after peptide addition (from 3.7 to ca. 1.3), which means that the monolayer became less organized. During the adsorption kinetics (up to 6 h), the bands had their intensity decreased owing to the lower number of molecules under the incident beam^34^, compared to the neat *S.e*.s. Typhimurium IM monolayer. As for the symmetric (2885 cm^−1^) and asymmetric (2965 cm^−1^) CH_3_ bands, lipid removal induced the chains lipid tilting becoming less exposed to the light beam, decreasing the bands intensity. After monolayer compression, the number of molecules reached by the incident infrared beam increased, thus yielding an increased band intensity, but not as high as for neat *S.e*.s. Typhimurium IM monolayer.

**Figure 3:**
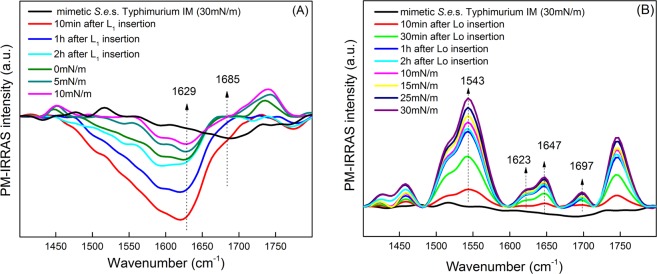
PM-IRRAS spectra for the monolayer mimicking the *S.e*.s. Typhimurium IM, in the absence and presence of L_1_ (**A**) and Lo (**B**) at 0.071 μM, under various conditions. The labels and procedures to take the spectra are the same of Fig. 2. The main difference is the region of the spectrum, which corresponds to the amide I and II regions (1500–1750 cm^−1^) for the peptides.

Amide I and amide II bands measured with PM-IRRAS for the peptides are shown in Fig. 3. These bands arise mainly from hybridized C=O and N-H vibrations from the peptide backbone, as for typical long chain proteins. However, their positions are not an indicative of Lo secondary structure, since this peptide presents a cyclic structure and has no freedom to adopt traditional structures like α-helices or β-sheets (Fig. 3B). On the other hand, the behavior of its linear analogue L_1_ can still be described in terms of its secondary structure (Fig. 3A). The spectra show the 1629 cm^−1^ and 1685 cm^−1^ vibrational bands at 10 min of L_1_ adsorption kinetics, which are related to amide I vibrational modes^22^. At 10 mN/m, it only depicts the band at 1629 cm^−1^. These bands are better seen when deconvoluted using Lorentz functions, as shown in the Supplementary Information. We can resort to the orientation function calculations in Barbosa *et al*.^22^, and then infer the proportion of each secondary structure for L_1_ interacting with the *S.e*.s. Typhimurium IM monolayer. Thus, The conformation of L_1_ is practically of random coils, however, in surface pressure lower than 10 mN/m there was an estimated 2% of antiparallel β-sheets, according to the band deconvolution analysis (obviously, this difference is within the dispersion of the experimental data). In comparison with the L_1_ Gibbs monolayer^22^, the interaction with the *S.e*.s. Typhimurium IM monolayer induced conformational changes in L_1_, with an increase in the proportion of random coils and the vanishing of the β-sheets. Moreover, all bands point downward, which means that the transition dipole modes of the bands are parallel to the monolayer surface. As for Lo, the symmetric shape of the peptide ring hampers any attempt to determine its orientation at the interface.

Interestingly, if one compares the interaction between the two peptides with *S.e*.s. Typhimurium IM and *S. aureus* monolayers (from the previous study^22^), the spectral characteristics (and consequently orientation and secondary structure composition) of the linear peptide (L_1_) are drastically changed, while for the cyclic Lo they are similar for both lipid compositions. L_1_ had its conformation changed to α-helices mostly upon interacting with *S. aureus* monolayer (55% DOPG and 45% CL), while it is mainly in random coils and β-sheets in the *S.e*.s. Typhimurium IM monolayer. This is clear evidence of how the secondary structure of a peptide may depend on the monolayer lipid composition.

### Membrane permeability assays

**Figure 4:**
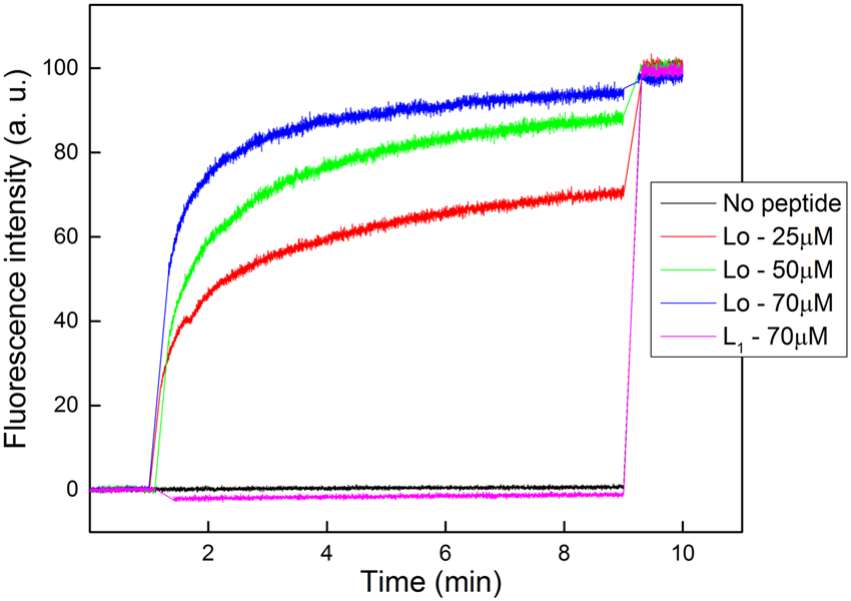
Kinetics of leakage for CF encapsulated in LUVs in response to L_1_ and Lo peptide addition at different concentrations. The vesicles simulating *S.e*.s. Typhimurium IM (78% DOPE, 4% CL, and 18% DOPG) were prepared at a concentration of 15 mM by extrusion, using a polycarbonate porous membrane to render vesicles of 100 nm in size. The removal of free CF outside the vesicles was performed through size-exclusion chromatography (Sephadex G-50 column) using 30 mM HEPES buffer, pH 7.4, with 100 mM NaCl. The fluorescence emission of CF was monitored at λ = 517 nm with excitation at λ = 492 nm (slit widths 5 nm). The peptides were injected 1 min after the kinetic measurements started. After 9 min, Triton X-100 (1%) was added to induce complete leakage of CF. Different peptide concentrations (Lo and L_1_) were added to the CF-LUVs suspension up to 70 μM.

It is known that AMPs act by disrupting or permeating bacterial membranes, which causes leakage in the microbe cell^36–39^. Interacting with monolayers does not mean that a drug or peptide will be able to permeate a cell membrane. We have therefore performed experiments to evaluate the peptide ability to permeate vesicles, whose lipid composition mimics *S.e*.s. Typhimurium IM, containing CF fluorescent dyes encapsulated. Figure 4 shows the results where LUVs with no peptides were used as a control, for which a low fluorescence intensity was observed due to the self-quenching of CF, being therefore used as 0% of leakage (no peptide). The presence of L_1_ peptide did not promote leakage even at high concentrations (70 µM), but it aggregated suppressing the fluorescence signal slightly^40^. Hence, the peptide was not able to perform the same activity on the vesicles (Fig. 4A), though the monolayer results suggested the removal of lipids. The difference in behavior may be due to the structure of the vesicle or due to the differences on the lipid packing. Anyway, this result could explain the lack of activity by L_1_ against *S.e*.s. Typhimurium, since the peptide is not able to permeate the vesicles. On the other hand, Fig. 4B indicates that Lo induced a concentration-dependent CF release from LUVs reaching 95% at 70 µM of Lo. Lo is thus able to permeate the membrane causing leakage, but to a lower extent compared to the damage caused in LUVs of S*. aureus* lipids^22^. It is consistent with the Langmuir monolayer data, confirming Lo affinity and insertion in the *S.e*.s. Typhimurium IM.

These permeability assays confirm that the cyclic structure of a peptide, such as Lo, does not impair the disruption of a bacterial membrane, in spite of its high conformational restriction. This conclusion is supported by other cases in the literature. For example, θ-defensin^41^, a cyclic peptide extracted from leukocytes of rhesus macaques and baboons, disrupts membranes via a carpet-wormhole mechanism^42^. The cyclic bactenecin permeates LUVs made with a mixture of phosphatidylcholine and phosphatidylglycerol^43^, and Tachyplesin I permeates bacterial as well as artificial lipid membranes^44^.

### LPS Langmuir Monolayers

**Figure 5:**
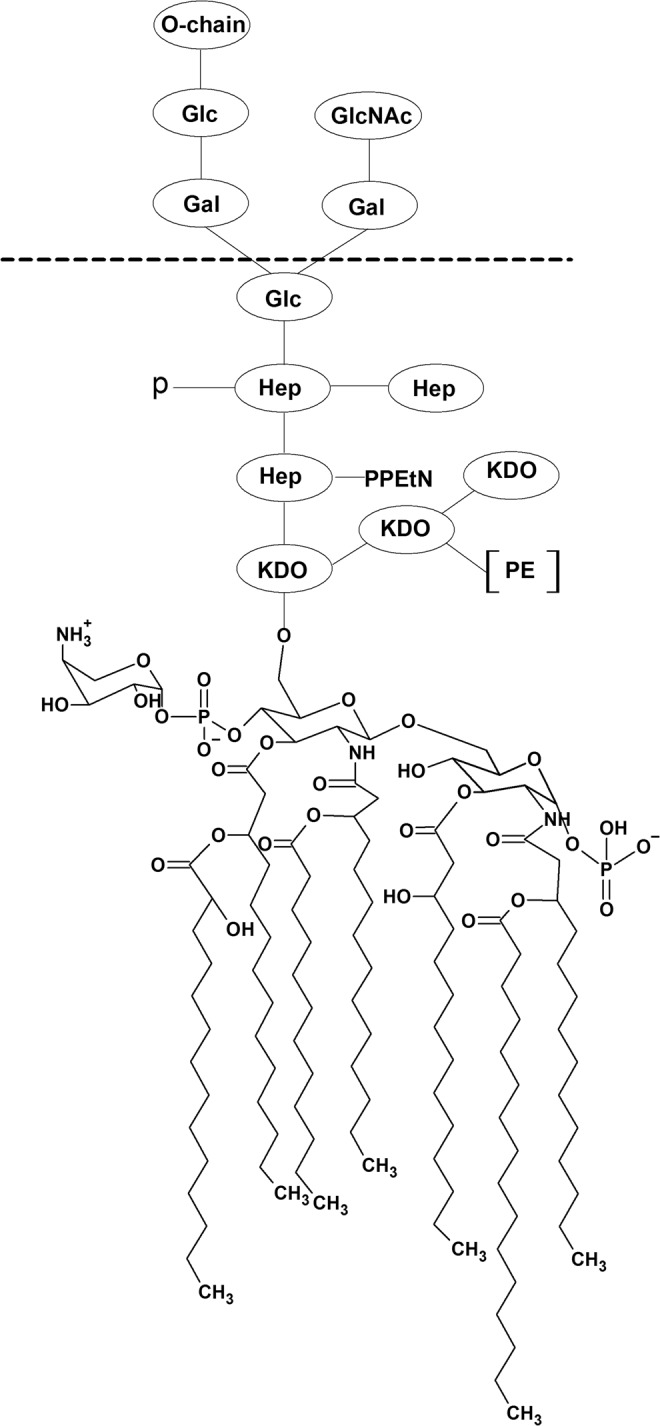
LPS structure from *Salmonella enterica* Senovar Typhimurium CS093 (reproduced with permission from Nobre *et al*.^57^). Abbreviations: Glc, glucose; GlcNAc, N-acetylglucosamine; Gal, galactose; Hep, heptose; KDO, 2 keto-3-deoxyoctulosonic acid; PE, phosphoethanolamine; PPEtN, pyrophosphoethanolamine.

Since Labaditin (Lo) is able to permeate LUVs simulating *S.e*.s. Typhimurium IM, but cannot exert biological activity against *S.e*.s. Typhimurium living cells, we extended our study to investigate the interaction between Lo and the outer leaflet of the outer membrane. This OM contains mainly lipopolysaccharides (LPS) that act as a barrier^45,46^ as can be inferred from its structure depicted in Fig. 5. Lipid A is negatively charged due to phosphate groups, the conserved region of LPS, responsible for the toxic effects^47^. The oligosaccharide core in Fig. 5 comprises a short sugar chain (up to 15 sugar residues) that connects lipid A to O-antigen. The latter component, the O-antigen, confers variety to LPS that may change among bacteria species, as it contains different types of sugar^46,48^.

**Figure 6:**
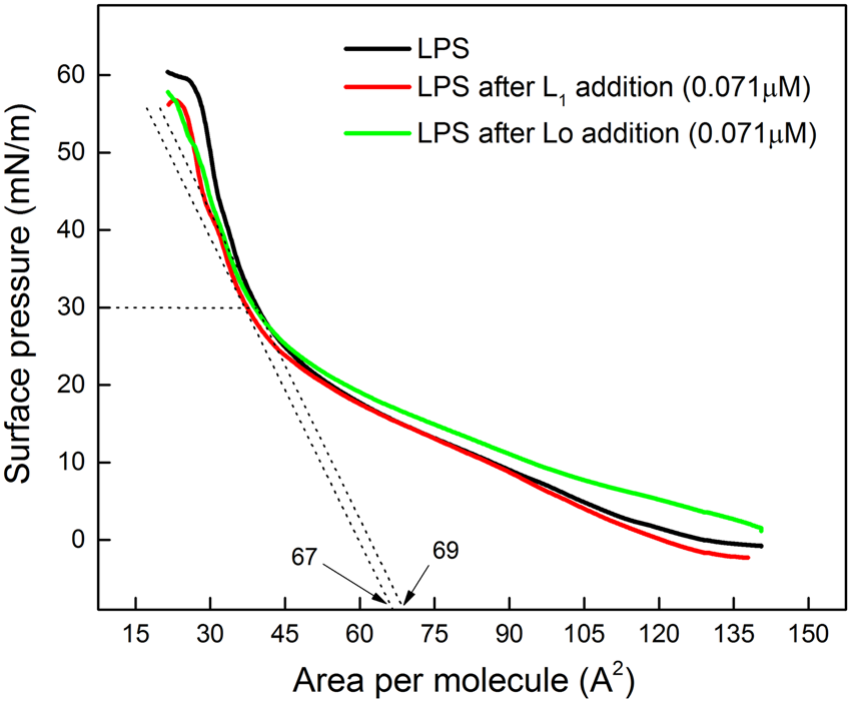
Surface pressure-area isotherms of neat LPS extracted from *S.e*.s. Typhimurium OM, and containing 0.071 µM of either Lo or L_1_. After LPS spreading at the air/water interface, the isotherm was obtained by compression with the trough barriers (10 cm^2^.min^−1^). For the isotherms containing peptides, the latter were injected 15 min after LPS spreading. No significant changes in the isotherms were induced by either Lo or L_1_.

The surface pressure-area isotherms for neat LPS extracted from *S.e*.s. Typhimurium OM, also including results for subphases containing 0.071 µM of Lo and L_1_, are shown in Fig. 6. The peptides Lo and L_1_ did not affect the LPS monolayer to any significant extent. This also applies to the monolayers at 30 mN/m, which corresponds to the lipid packing in cell membranes.

**Figure 7:**
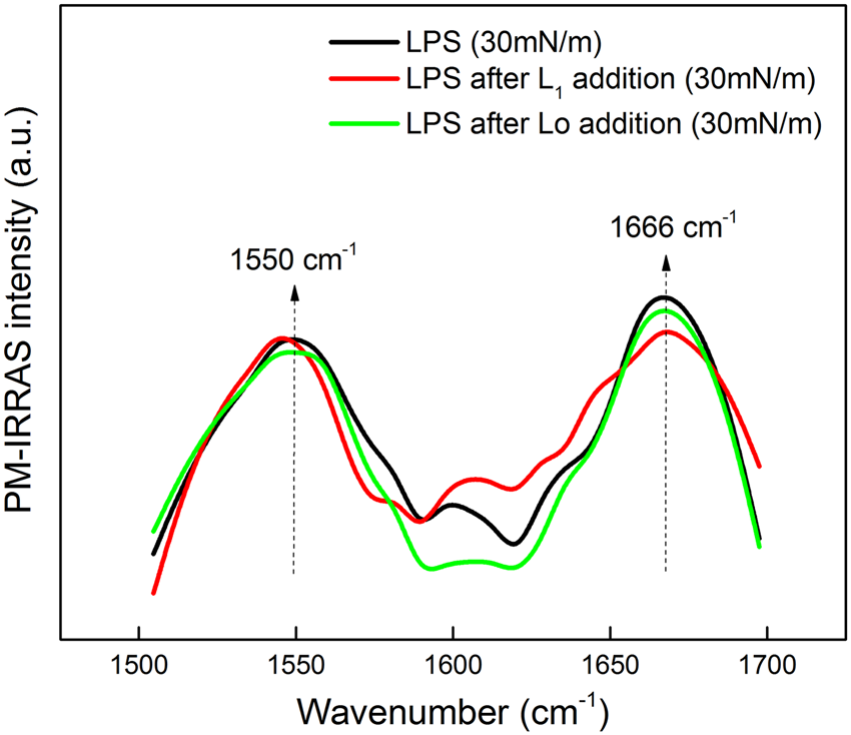
PM-IRRAS spectra in amide I and II regions (1500–1750 cm^−1^) for the monolayer of neat LPS extracted from *S.e*.s. Typhimurium OM, and upon addition of L_1_ and Lo, at 30 mN/m. The monolayer was obtained by spreading LPS and then waiting 15 min for evaporation of the spreading solvent. Monolayer compression was carried by closing the trough barriers at 10 cm^2^.min^−1^. No significant differences are seen in the spectra, which could be induced by incorporation of either L_1_ or Lo.

The amide I and amide II bands in the PM-IRRAS for neat LPS and the peptides at 30 mN/m are observed at 1666 cm^−1^ and 1550 cm^−1^, respectively, in Fig. 7. They should arise from LPS^49^, as can be inferred from the structure in Fig. 5. Similarly to what was observed in the surface pressure isotherms, no significant effects are noted upon incorporating either Lo or L_1_ onto the LPS monolayer.

This lack of effect by the peptides is in sharp contrast to the changes they induced in the *S.e*.s. Typhimurium IM monolayer in Fig. 1. It confirms that the LPS wall is an important physical barrier, which would also explain the lack of bactericide activity of Lo – the latter can permeate the lipid inner membrane but not the LPS layer. This is in line with reports in the literature according to which the LPS outer layer protects bacteria against some harmful molecules, such as AMPs^50,51^. The strategic position of the O-antigen in the outermost portion of LPS hides the negative charged lipid A from electrostatic interaction with the cationic antimicrobial peptides. In *S.e*.s. Typhimurium, lipid A contains an additional fatty acid which decreases the negative character of the bacterial membrane. Moreover, modifications are performed in the anionic phosphate groups of lipid A and core region, through addition of cationic aminoarabinose and zwitterionic phosphoethanolamine. Also, there may be proteolytic degradation of antimicrobial peptides by outer-membrane proteases from *S.e*.s. Typhimurium^52^. Indeed, *S.e*.s. Typhimurium contains additional fatty acid and substituent groups in comparison to *E. coli*^53^, which make *S.e*.s. Typhimurium more resistant to antimicrobial peptides^52,54^.

## Conclusions

With a series of monolayer and large unilamellar vesicles (LUVs) experiments, we could determine the reason why the cyclic peptide Labaditin (Lo) is not efficient in killing *S.e*.s. Typhimurium. Lo and its linear analogue L_1_ caused large changes in the *S.e*.s. Typhimurium IM monolayer, which were verified in surface pressure isotherms and PM-IRRAS spectra. These molecular-level interactions in monolayers did not translate entirely for the environment of LUVs, since only Lo was capable of permeating the vesicles representing the *S.e*.s. Typhimurium IM, inducing leakage. It seems that the difference between L_1_ and Lo is of the same nature as observed for LUVs made with a lipid composition representing *S. aureus*^22^; indeed, unlike L_1_, Lo did cause leakage in the vesicles and this correlated with its activity against *S. aureus*. On the basis of the monolayer and LUV studies with the lipid composition of *S.e*.s. Typhimurium IM, one could therefore predict that Lo would exhibit bactericide activity as it did for *S. aureus*. However, the lack of activity is explained by the finding that Lo did not affect the monolayer of an LPS extract of *S.e*.s. Typhimurium OM. This may be due to the highly packed molecular arrangement in the LPS layer, which was indeed apparent in the surface pressure isotherm studied here, and may be attributed to the high density of sugars and charges in the LPS from *S.e*.s. Typhimurium. In principle, the lack of interaction and activity should not be ascribed to the cyclic nature of Lo since the cyclic Polymyxin B^55^, derived from bacteria *Bacilluspolymyxa*, was proven to act against Gram-negative bacteria by binding to their outer LPS layer^55,56^.

An important implication of our work is related to the need of assessing distinct types of cell membrane models to be able to infer any correlation with the bactericide activity of a peptide or drug. In fact, if we were to take the changes induced in the monolayer for the lipid component of the *S.e*.s. Typhimurium IM, we would be completely deceived. The immense changes in surface pressure isotherms and PM-IRRAS data caused by Lo and L_1_ found no correspondence on the results from the bactericide assays. Likewise, the leakage caused by Lo in LUVs mimicking *S.e*.s. Typhimurium IM did not signify bactericide activity. In conclusion, for Gram-negative bacteria, such as *S.e*.s. Typhimurium, any attempt to correlate results from membrane models and activity must consider the LPS outer layer.

## Materials and Methods

### MIC determination

The peptides Lo and L_1_ were obtained from Aminotech Research ( > 95% purity). *Salmonella enterica* Senovar Typhimurium (CS093) cells were cultured overnight in sterile Luria-Bertani (LB) broth without shaking, and at a late exponential phase (OD600 = 1.0) the culture was diluted to OD600 of 0.1 and used as inoculum. Microdilution was performed using a series of 200 µL of LB broth and containing two-fold serial dilution of one of the synthetic peptides (Lo or its linear counterpart L_1_), which were prepared in the 96-well microtiter plates. Approximately 10^4^ cells from the inoculum as described above were inoculated. The plates were incubated for 18 h at 37 °C, and the tests were performed in triplicate, according to Nobre *et al*.^57^.

### Langmuir monolayers

1,2-Dioleoyl-sn-glycero-3-phosphoethanolamine (DOPE), 1,2-dioleoyl-sn-glycero-3-phosphatidyl glycerol (DOPG) and 14:0 cardiolipin (CL) were purchased from Avanti Polar Lipids. For producing Langmuir monolayers, we simulated the *S.e*.s. Typhimurium IM using 78% DOPE, 18% DOPG, and 4% CL, according to the literature^4,5^. As mentioned in Barbosa *et al*.^22^, we do not neglect the importance of lipid composition, such as the presence of branched lipids, for the bacterial susceptibility against antimicrobial peptides. However, we decided to focus our efforts on determining the role of each membrane (IM and OM) from *S.e*.s. Typhimurium, and how it is affected by Lo and L_1_ taking into account that IM contains low concentration of anionic lipids^58,59^. The stock solution was prepared in chloroform:methanol (4:1 v/v). The subphases were prepared using Millipore Direct-Q ultrapure apyrogenic water (resistivity of 18.2 MΩ.cm at 25 °C), and the reagents were of the highest commercially available purity grade. The Lo and L_1_ solutions (150 µM) were separately prepared by diluting the peptide powder in ultrapure water. Surface pressure isotherms were measured in a mini-KSV Langmuir trough (KSV Instruments Ltd, Helsinki, Finland) equipped with a Wilhelmy plate made of filter paper, at 21 °C. The isotherms were obtained by spreading 50 µL of the lipid from a stock solution at 627 µM on the air/water interface. The removal of the spread organic solvent was spontaneous via self-evaporation along 15 min. Prior to the π-A isotherms, adsorption kinetics of the peptide at different concentrations were obtained on the lipid monolayer (at null surface pressure). The time dependence of the surface pressure was monitored to ensure adsorption had reached equilibrium before compressing the monolayer. Compression was carried out using two movable barriers at 10 cm^2^.min^−1^. Surface pressure isotherms were performed in triplicate, and the maximum error found was 3 Å^2^/molecule. Polarization-Modulated Infrared Reflection-Absorption Spectroscopy (PM-IRRAS) measurements were performed using a KSV PMI 550 instrument (KSV Instruments Ltd, Helsinki, Finland) in a mini KSV Langmuir trough. The light beam reached the monolayer at a fixed incidence angle of 81°, for which the upward-oriented bands indicate a transition moment preferentially parallel the surface plane, whereas downward bands indicate orientation perpendicular to the surface. All the experiments were carried out in a clean room at 21.0 ± 0.1 °C. The experimental setup was the same used above in Langmuir monolayers. In these PM-IRRAS experiments we used the highest peptide concentration analyzed in the Langmuir monolayer assays (0.071 μM) to amplify the band signal. Spectra were collected every 5 mN/m of surface pressure (monolayer compression) and sometimes during the adsorption kinetics of the peptide.

### Carboxyfluorescein (CF) release from LUVs

For the leakage assays, LUVs containing the phospholipid composition of *S.e*.s. Typhimurium IM were prepared at a concentration of 15 mM. The mixture of lipids was dried under a N_2_ stream and left in vacuum for 6 h to form a lipid film. First, multilamellar vesicles were obtained by mechanical stirring with a 30 mM HEPES buffer solution, pH 7.4, with 50 mM CF and 86 mM glucose added to adjust the solution osmolarity. The unilamellar vesicles were obtained by extrusion of the multilamellar suspension using a polycarbonate porous membrane, to render 100 nm size. This solution was eluted by size-exclusion chromatography through a Sephadex G-50 column to remove the free CF outside the vesicles, using 30 mM HEPES buffer, pH 7.4, with 100 mM NaCl. The CF-LUVs were collected in tubes, diluted and the phospholipid concentration was determined by phosphate analysis according to the methodology by Rouser *et al*.^60^. The fluorescence emission of CF was monitored at λ = 517 nm, with excitation at λ = 492 nm (slit widths 5 nm), using a spectrofluorimeter Cary Eclipse, Varian. Different concentrations of peptide (Lo and L_1_) were added to the CF-LUVs suspension. At the end of each experiment Triton X-100 (1% v:v) was added for the release of all CF. The percentage of CF leakage was calculated according to the equation: 100(Ft-Fo)/(Fmax-Fo), where Ft is the fluorescence at a given time, Fo is the initial fluorescence (before addition of peptide), and Fmax is the maximum fluorescence after addition of Triton X-100^61–63^. All experiments were performed in triplicate.

## Supplementary information

Supplementary Information

## Data Availability

All data generated or analyzed in this study are included in this published material and its Supplementary Information file. Raw data files (Origin 9.0) are available from the corresponding authors or first author on reasonable request.
