# Supplementary material for: The cyclic peptide labaditin does not alter the outer membrane integrity of Salmonella enterica serovar Typhimurium
Source: Sci Rep. 2019 Feb 13;9:1993. doi: 10.1038/s41598-019-38551-5 (PMC6374527; doi:10.1038/s41598-019-38551-5)
Supplement: Supplementary file 1 — Supplementary Information [file 41598_2019_38551_MOESM1_ESM.docx]

**Supporting Information**

# The cyclic peptide labaditin does not alter the outer membrane integrity of *Salmonella enterica* serovar Typhimurium

# *Simone C. Barbosa^1^*, Thatyane M. Nobre^1^, Diogo Volpati^2^, Eduardo M. Cilli^3^, Daniel S. Correa^4^, Osvaldo N. Oliveira Jr^1^**

# ^1^São Carlos Institute of Physics, University of São Paulo, CP 369, 13560-970, São Carlos-SP, Brazil.

^2^Sol Voltaics AB, 223 63 Lund, Sweden.

^3^Institute of Chemistry, IQ-UNESP, 14800-060, Araraquara-SP, Brasil.

^4^Nanotechnology National Laboratory for Agriculture (LNNA), Embrapa Instrumentação, 13560-970, São Carlos, SP, Brasil.

**Figure SI.1** - PM-IRRAS spectra taken at the air/water interface: (a) for the pure monolayer mimicking the *S.e*.s. Typhimurium IM, at 30mN/m; (b) 10 min after L_1_ injection in the *S.e*.s. Typhimurium IM monolayer (at 0mN/m); (c) for S.e.s. Typhimurium IM monolayer after L_1_ adsorption, compressed at 10 mN/m. The concentration of L_1_ was 0.071μM. The region of the spectra shown corresponds to the amide I and II regions (1500–1750 cm^−1^) from the lipids.
